# Protocol for Fecal Microbiota Transplantation in Inflammatory Bowel Disease: A Systematic Review and Meta-Analysis

**DOI:** 10.1155/2018/8941340

**Published:** 2018-09-13

**Authors:** Haiming Fang, Lian Fu, Jiajia Wang

**Affiliations:** ^1^Department of Gastroenterology and Hepatology, The Second Hospital of Anhui Medical University, Hefei, Anhui Province, China; ^2^Department of Pharmacology, Anhui Medical University, Hefei, Anhui Province, China

## Abstract

**Background:**

Fecal microbiota transplantation (FMT) is an emerging treatment approach for inflammatory bowel disease (IBD). The donor selection, the separation of fecal bacteria, the frequency of FMT, the way of infusion, the long-term safety, and efficacy are still uncertain.

**Aim:**

To further study the efficacy and safety and protocol of FMT for IBD.

**Methods:**

A systematic review and meta-analysis were conducted until February, 2018. Clinical remission was established as the primary outcome.

**Results:**

A total of 596 paediatric and adult IBD patients were enrolled, and 459 patients received FMT therapy. 28.8% (132/459) patients achieved clinical remission during follow-up. 53% (241/459) patients achieved clinical response. The pooled estimated clinical remission for ulcerative colitis (UC) was 21% (95% CI: 8%-37%) and 30% (95% CI: 11%-52%) for Crohn's disease (CD), both with a risk of heterogeneity; 10% (95% CI: 0%-43%) for paediatric UC; 26% (95% CI: 10%-48%) for adult UC; 45% for paediatric CD (95% CI: 24%-66%); 22% (95% CI: 3%-52%) for adult CD. Meta-analysis of cohort studies showed that moderate-severe IBD patients could achieve more significant remission from FMT than mild-moderate patients (*P*=0.037). Delivery route has no impact on the efficacy of FMT in UC and CD. Based on current available evidence, a trend was observed towards higher clinical remission rate of frozen stool FMT than that of fresh stool for UC, while there was no significant difference between fresh and frozen FMT for CD. The optimal donor stool for FMT is still uncertain. Meta-analysis of RCTs showed that FMT treatment achieved significantly higher clinical remission rate than placebo for UC (28% versus 9%,* P*=0.0003).

**Conclusion:**

FMT is an effective and safe therapy for both paediatric and adult IBD; fresh or frozen donor stool, delivery route, and antibiotic pretreatment or not have no impact on the efficacy of FMT in IBD. FMT might be a potential rescue therapy and even an initial standardized therapy for IBD. However, few data exist on long-term safety and efficacy and further validation is needed.

## 1. Introduction

The gut microbiota provides an intestinal biological barrier against pathogens and has a pivotal role in the maintenance of intestinal homeostasis and modulation of the host immune system [1]. Multifaceted microbial population is considered as a human organ with critical function. The specific changes in the composition of gut microbiota, termed dysbiosis, have been associated not only with many gastrointestinal (GI) diseases but also with metabolic diseases, autoimmune diseases, allergic disorders, and neuropsychiatric disorders [2].

Inflammatory bowel disease (IBD) is a chronic relapsing gastrointestinal tract inflammatory disorder, including ulcerative colitis (UC) and Crohn's disease (CD), which is characterized by progressive transmural inflammation of the gut. Over the past decades, the incidence and prevalence of IBD have gradually increased in Asian countries, including China. Although the etiology of the disease remains unclear, IBD involves interactions among genetic susceptibility, environmental factors (antigens derived from commensal bacteria), intestinal microbiota, and the immune system [3]. Several clinical and preclinical researches have shown that dysbiosis of gut microbiota is associated with IBD, which seems to play a crucial role in the development of this disease [4, 5].

Manipulating the gut microbiota via fecal microbiota transplantation (FMT) is an emerging treatment approach. The concept of FMT was first described in China during 4th century. Human fecal suspension by mouth was used to treat patients who had food poisoning or severe diarrhea [6]. FMT was reported in 1958 for treating refractory and recurrent Clostridium difficile infection (RCDI) [7]. Recent studies have shown that FMT is an effective treatment for RCDI, with greater than 90% success rate and can be considered as an antibiotic replacement for RCDI [8]. Due to the satisfactory effect of FMT for RCDI, the American Society of Gastroenterology, the European Association of Clinical Microbiology, and European Association of Infectious Diseases had listed FMT in the treatment guidelines of RCDI in 2013 and 2014, respectively [9, 10]. This has encouraged researches on FMT as a potential therapy for other microbial-related diseases such as IBD.

Since Bennet [11] reported the first case of FMT for the treatment of UC in 1989, lots of case reports, case series, and, more recently, randomized controlled trials (RCTs) had been reported regarding investigating the efficacy and safety of FMT for IBD. Sun et al. [12] reported that the rate of clinical remission in patients with UC was 30.4%. Shi et al. [13] showed that 41.58% patients with UC achieved clinical remission and 65.28% achieved clinical response. Moayyedi et al. [14] and Paramsothy et al. [15] showed that the rate of clinical remission in patients who received FMT was higher than that of placebo. However, Rossen et al. [16] was not able to demonstrate a significant difference in the rate of clinical remission between the FMT and placebo groups.

Up to date, a number of questions on the protocol of FMT remain unanswered. The donor selection, the separation of fecal bacteria, the frequency of FMT, the way of infusion, the long-term safety, and efficacy are still uncertain. Many reports recommend giving bowel lavage before donor feces infusion and also giving antibiotics before the transplantation to facilitate the colonization of microbiota from the donor [17]. However, whether colonic lavage and/or antibiotic therapy have any impact on FMT for IBD is still uncertain. There is also uncertainty as to whether fresh or frozen fecal samples should be used for FMT in IBD. We performed a systematic review and meta-analysis with the most up-to-date and reliable evidence to assess these questions.

## 2. Materials and Methods

### 2.1. Inclusion Criteria

Studies included in this systematic review must report on clinical efficacy and safety of FMT for IBD in paediatric or adult subjects. Only cohort studies and RCTs were enrolled for meta-analysis. Literature language was limited to Chinese or English.

### 2.2. Exclusion Criteria

Studies were excluded if data were only case reports or case series of FMT for IBD. Studies were also excluded if data had no clear definition of remission or clinical endpoints or they only included patients who had coinfection with Clostridium difficile or other pathogens. In addition, systematic review and meta-analyses data were also excluded.

### 2.3. Literature Search Strategy

A systematic review was performed in accordance with the PRISMA [42], Cochrane [43], and MOOSE [44] guidelines. We searched Medline (from 1948), web of science (from 1926), Cochrane library (for all years), EMBASE (from 1947), CNKI, Wanfang Data, and Pubmed. Study types included randomized controlled trials (RCTs), cohort studies and case studies (case series and case reports), nonrandomized controlled studies, and nonrandomized experimental studies. We also searched proceedings from annual world meetings including European Crohn's and Colitis Organization (ECCO), Digestive Diseases Week (DDW), American College of Gastroenterology (ACG) and Advances in IBD (AIBD), and United European Gastroenterology Week (UEGW) from 1995 to February, 2018. The search key words were “ulcerative colitis” or “UC” or “Crohn disease” or “CD” or “inflammatory bowel diseases” or “IBD” or “adult IBD” or “paediatric IBD” or “child IBD ” AND “fecal microbiota transplantation” or “fecal microbiota transfusion” or “stool microbiota transplantation” or “stool microbiota transfusion” or “faecal microbiota transplantation” or “gut flora” or “intestinal flora”.

Literature screening, data extraction, and reviewing work were accomplished by two investigators in strict accordance with the inclusion and exclusion criteria. In case of dispute, the key decision was made by the third investigator, who is a famous statistical expert.

### 2.4. Study Quality Assessment

For eligible cohort studies, we used the Newcastle-Ottawa Scale [45] on the standard 9-point scale to assess the methodological quality, including the study population selection, comparability, and outcome evaluation. Thus the study was divided into the following items: the selection of groups (for age, disease severity), ascertainment of FMT treatment and absence of prior exposure to FMT, outcome quality evaluation (predefined disease activity index score or mucosal evaluation), and adequate follow-up time and reports. Follow-up time ≥ 3 months was considered to be abundant follow-up; there were three studies indicating no sufficient follow-up reports (≥ 1 month). Through comprehensive analysis and evaluation, the studies quality was moderate to high. The Cochrane risk of bias score was used to assess bias in RCTs [46], incorporating random sequence generation, blinding, allocation concealment, selective reporting, and incomplete outcome data.

### 2.5. Statistical Analysis

Descriptive statistics were performed on data extracted from all included studies. Efficacy of FMT in IBD was evaluated as clinical remission (primary outcome) or clinical response defined by the respective study authors. Safety was assessed using reported adverse event and serious adverse event data. For cohort studies, the pooled proportion of achieving clinical remission rates and subgroup analyses were analyzed using R software. The pooled effect sizes and 95% confidence intervals were obtained using the random effects model. For meta-analyses of RCTs, a random effects model was selected to assess the pooled estimate of clinical remission using Review Manager 5.2 (Nordic Cochrane centre, Copenhagen, Denmark, 2012), the pooled odd ratios (P-ORs) were calculated by weighting individual ORs by the inverse of their variance.* P* value < 0.05 was considered statistically significant. The Cochran's Q test (P value < 0.10) and Higgins' test [I^2^] (low heterogeneity: <25%, moderate heterogeneity: 25-75%, high heterogeneity: >75%) were used to assess the statistical heterogeneity [47]. To evaluate the stability of our results, sensitivity analyses were performed. Subgroup analyses were performed according to IBD subtype (UC versus CD), preparation of inoculum (fresh versus frozen), population (paediatric versus adult), and route of administration (upper versus lower gastrointestinal tract). Funnel plots and Egger's test were used to assess potential publication biases.

## 3. Results

### 3.1. Literature Screening Process

**Figure 1:**
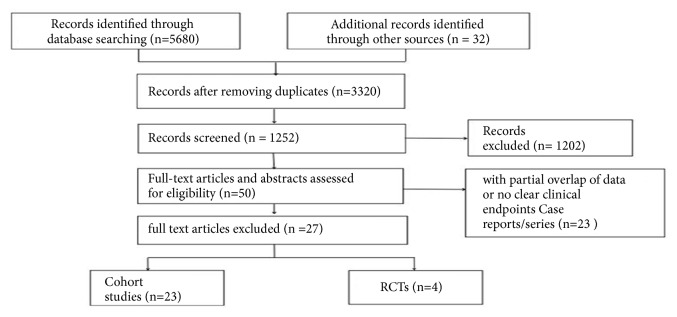
Literature screening process and flow diagram.

A total of 5680 articles were identified in the search, which included 3320 records after removing duplicates. Finally a total of 27 articles of FMT for IBD were deemed to satisfy the study selection criteria (as shown in Figure 1) including 23 cohort studies (21 uncontrolled, 2 controlled) and 4 randomized controlled trials (RCTs). A total of 596 IBD patients were enrolled in which 459 patients received FMT therapy, and 132 IBD patients achieved clinical remission during follow-up.

In this system review, both paediatric and adult IBD were included. Age ranged from 7 to 71 years and follow-up time ranged from 1 to 72 months. Severity of disease varied from mild to severe (as shown in Table 1).

### 3.2. Meta-Analysis of Cohort Studies

In strict accordance with the inclusion and exclusion criteria, 23 cohort studies were deemed to satisfy the selection criteria including 15 in UC, 4 in CD, and 4 in both UC and CD with a total of 319 patients (as shown in Table 1), which were all treated with FMT and the efficacy and safety were assessed during the long enough follow-up.

23 cohort studies included 319 IBD patients (225 UC and 94 CD), in which 129 patients had mild or moderate disease, 55 patients moderate-severe, 67 patients “refractory”, 36 patients “active disease” and 32 patients were hormone-dependent. Studies included both adults (252 cases) and paediatric (67 cases) patients. 213 patients were treated with fresh donor stool and 80 patients were treated with frozen stool protocol, and in 26 patients the donor of fecal microbiota was not identified.

FMT was administered via the upper gastrointestinal tract (nasogastric/nasojejunal tube or endoscopic duodenal infusion) in 105 patients, via the lower gastrointestinal tract (enema, colonoscopy or rectal tube) in 157 patients, and via both routes in 57 patients (as shown in Table 1).

Donor stools were mainly from healthy adults or children family members or close friends or volunteers. Among the 319 patients, 134 patients received fecal bacteria from their family members or close friends in which 26 patients (21%, 26/134) achieved clinical remission. 79 patients received fecal bacteria from healthy anonymous donors with 37% (29/79) patients achieving clinical remission, 37 patients received fecal bacteria from family members or friends and healthy volunteers, and 69 patients had no donor source report (as shown in Table 1).

#### 3.2.1. Pooled Remission and Response of FMT for IBD

Among the 23 cohort studies, including 319 IBD patients, 93 patients achieved clinical remission; meta-analysis showed that the pooled proportion of clinical remission of FMT in IBD was 27%, with a high heterogeneity (as shown in Table 2 and supplement Figure 1).

#### 3.2.2. Ulcerative Colitis versus Crohn's Disease

The 319 patients consisted of 225 UC patients and 94 CD patients. Among the 225 UC patients, 53 cases achieved clinical remission during follow-up; the pooled estimate of clinical remission rate was 21%, with a high risk of heterogeneity after meta-analysis. Among the 94 CD patients, 40 patients achieved clinical remission; the pooled proportion of clinical remission was 30%, with a risk of heterogeneity (as shown in Table 2 and supplement Figure 2). Publications bias was observed in this meta-analysis.

#### 3.2.3. Paediatric versus Adult Populations

Totally, 67 paediatric IBD (47 UC and 20 CD) and 252 adult IBD (178 UC and 74 CD) were enrolled in the subgroup analysis. 6 patients in 47 paediatric UC achieved clinical remission, and 45 patients in 178 adult UC achieved clinical remission. Meta-analysis showed that the pooled proportion of clinical remission rate of FMT for paediatric UC was 10%, and 26% for adult UC, both with a high risk of heterogeneity (as shown in Table 2).

9 patients in 20 paediatric CD achieved clinical remission and 31 patients in 74 adult CD achieved clinical remission. The pooled estimate of clinical remission rate of FMT for paediatric CD was 45% and 22% for adult CD (as shown in Table 2).

Up to date, no randomized controlled trials were published assessing FMT in paediatric IBD.

#### 3.2.4. Preparation of Inoculum

The subgroup analysis of all cohort studies showed that preparation of inoculum and route of administration might play a significant role in clinical remission.

Among the 225 UC patients, 164 patients accepted fresh donor stool FMT in which 31 cases achieved clinical remission, and 35 patients accepted frozen stool FMT in which 13 cases achieved clinical remission. The pooled clinical remission rate was 15% of fresh FMT for UC and was 42% of frozen FMT for UC (as shown in Table 2). Additionally, 26 cases in the 225 UC were unclear whether fresh or frozen FMT.

For CD patients, 49 patients accepted fresh donor stool FMT in which 24 cases achieved clinical remission, and 45 patients accepted frozen donor stool FMT in which 16 cases achieved clinical remission. The pooled clinical remission rate was 36% of fresh stool FMT for CD and was 28% of frozen stool FMT for CD (as shown in Table 2).

#### 3.2.5. Route of Administration

Among the 225 UC patients, 137 patients received FMT via the lower gastrointestinal tract in which 38 cases achieved clinical remission, and 42 patients via the upper gastrointestinal tract in which 6 cases achieved clinical remission. The pooled proportion of clinical remission rate of upper gastrointestinal tract FMT for UC was 8% and 31% of lower gastrointestinal tract FMT for UC. Additionally, 46 UC patients obtained both upper and lower routes FMT (as shown in Table 2).

Among CD patients, 63 patients received FMT via upper gastrointestinal delivery route in which 26 cases achieved clinical remission and 20 patients received lower gastrointestinal route FMT in which 10 cases achieved clinical remission. The pooled proportion of clinical remission rate of upper gastrointestinal tract FMT for CD was 23% and 27% of lower gastrointestinal tract FMT (as shown in Table 2).

#### 3.2.6. Meta-Analysis of Disease Severity and Preantibiotic

Among the 319 patients (UC and CD), 129 patients were described as mild-moderate disease in which 27 cases achieved clinical remission, and 133 patients were defined as moderate-severe disease including 67 refractory patients and 32 hormone-dependent patients in which 45 cases achieved clinical remission. Overall, the pooled proportion of clinical remission rate was 20% of FMT for mild-moderate IBD and was 30% for the moderate-severe IBD (as shown in Table 2). Moderate-severe IBD patients seem to benefit more from FMT treatment than mild-moderate patients significantly (*P*=0.037).

7 cohort studies assessing 85 patients including 69 UC and 16 CD achieved pretreatment with antibiotics prior to FMT. Totally, 29 patients achieved clinical remission and the pooled proportion of clinical remission rate was 25%. The 234 patients without antibiotic pretreatment included 156 UC and 78 CD, in which 64 patients achieved clinical remission after FMT; the pooled estimate clinical remission rate was 28% (as shown in Table 2).

### 3.3. Meta-Analysis of Randomized Controlled Trials

There were four RCTs of FMT in UC (Table 3), which included a total of 277 patients; 140/277 (51%) patients received donor FMT and 137/277 (49%) received placebo. The placebo was autologous stool or water; the ratio of FMT versus placebo was 1 : 1. Enrolled patients in the four RCTs were active and mild-moderate UC. Delivery method included colonoscopy followed by enema [15, 41], enema [14], or nasoduodenal tube [16]. Follow-up time ranged from 7 to 12 weeks.

In meta-analysis of the 4 RCTs, the clinical remission was achieved in 39 of 140 (28%) patients in the FMT groups compared with 13 of 137 (9%) patients in the placebo groups. The OR was 3.67 (95% CI: 1.82-7.39, P=0.0003), with no statistically significant heterogeneity between studies (I^2^=0%) (as shown in supplement Figure 3).

Clinical response was achieved in 69/140(49%) patients who received donor FMT and 38/137 (28%) patients achieved response with placebo (OR: 2.48, 95%CI: 1.18-5.21); heterogeneity was moderate (I^2^ = 52%). FMT showed more significant benefits of induction remission in UC than placebo (as shown in supplement Figure 3).

### 3.4. Microbiological Analyses

At present, there are few studies on the analysis of intestinal microbial community. Most of the studies mainly compared the changes of flora in the fecal samples before and after transplantation and identified the flora by extracting bacterial DNA or sequencing 16s rRNA from stool samples [30]. A range of studies commented on recipient microbiota changes after FMT treatment with increased diversity or richness [29, 34, 38]. Most studies suggested that the changing tendency of flora after FMT tended to the donor. Cui et al. [21] found that most of the bacterial diversity were increased after FMT and showed a trend highly similar to their related donors. Bacterial diversity of a patient was reduced after FMT, but finally it was confirmed that the patient was given antibiotics before and after FMT in this study, and the author considered that the use of antibiotic affected the diversity and composition of fecal microbiota greatly. Brittnacher et al. [37] showed further proof of similarity with the donors appearing in a range from 2-12 weeks, and the largest similarity was 69%, but whether it is consistent with clinical remission is not yet conclusive. Moayeddi et al. [14] found that there was a statistically significant change in microbiota composition with more diversity in the treatment group compared with the placebo group. Donor with a significant microbial enrichment for the family* Lachnospiraceae* and the genera* Ruminococcus* was associated with successful FMT; there was a trend for responders having microbiota that was more similar to the donor than nonresponders, but this did not achieve statistical significance differences. Some studies identified that therapeutic success of FMT in UC had correlation with increased donor microbial diversity. Paramsothy et al. [15] reported that recipient microbial diversity at baseline predicted response to FMT and microbial diversity increased with FMT during 8-week follow-up. Multidonor FMT batches used for the FMT infusions acquired substantially greater microbial diversity compared with the individual donors. The increase of microbial diversity in recipient after FMT in responders was greater than that in nonresponders.

### 3.5. Safety and Adverse Events

Each of the studies monitored and reported the adverse events after FMT. Majority adverse events were transient gastrointestinal complaints, such as diarrhea, abdominal pain, bloating, and borborygmus. Fever in varying degrees (with or without chills) was self-limited after FMT. And vomiting, nasal congestion, runny nose, or sore throat occasionally appeared. No serious adverse events were reported. Rare adverse events were also reported. Scaldaferri et al. [24] conducted FMT to eight mild-moderate UC patients; as a result, two patients had worse disease and one patient had unexplained kidney stones after FMT. Vaughn et al. [38] treated 19 CD patients with FMT; urticaria occurred in one patient and remitted after hormone therapy; another patient did not achieve clinical improvement and finally performed surgery eight weeks after FMT.

In general, FMT was well tolerable, safe, and effective in IBD. However, the follow-up time ranged from 1 to 72 months in the present study. There is insufficient data on the long-term efficacy and safety of FMT for IBD at present.

### 3.6. Sensitivity Analysis

**Figure 2:**
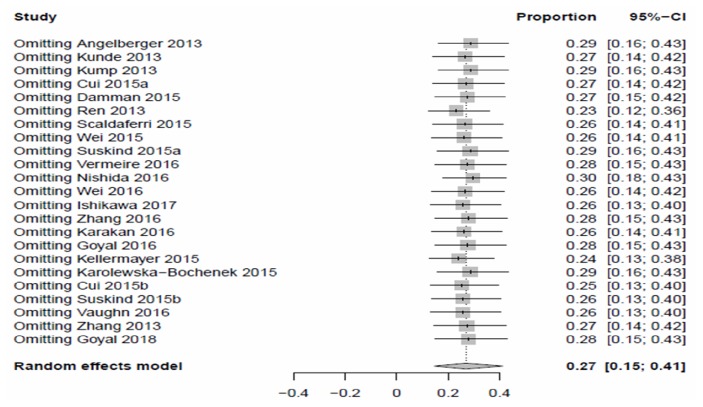
Forest plots of sensitivity analyses.

Sensitivity analyses were performed, and the results showed no significant change when any one study was excluded (as shown in Figure 2).

**Figure 3:**
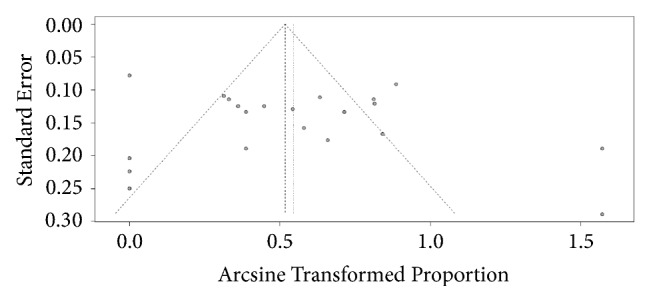
Funnel plots of publication bias.

Based on the funnel plots of publication bias, there was no evidence of publication bias (Egger's statistic = 0.78, p = 0.45) (as shown in Figure 3).

## 4. Discussion

### 4.1. FMT Is an Effective Therapy for IBD

This study represents an up-to-date meta-analysis study with systematic retrospective and complete cohort study and RCTs properties that can more critically evaluate the efficacy and safety of FMT on IBD. We identified a total of 596 IBD patients in which 459 patients accept FMT treatment; overall, 28.8% (132/459) of IBD patients achieved clinical remission, and 53% (241/459) of patients achieved clinical response during follow-up.

The treatment effects on patients who have undergone FMT to treat UC appear very promising, especially for patients with multiple infusions administered via the lower gastrointestinal tract. Colman et al. [48] reported a meta-analysis assessing only UC cohort studies, identified 79 patients with a pooled proportion achieving clinical remission of 22% [95% CI: 10.4%-40.8%] in 2014. In 2017, Paramsothy et al. [49] reported a meta-analysis that identified 24 UC cohort studies assessing 307 patients, with a pooled proportion of clinical remission rate of 33% [95% CI: 23%-43%], with a moderate risk of heterogeneity. However, this review primarily included some case reports as well as cohort studies and RCTs. The present meta-analysis primarily consisted of cohort studies and RCTs, and the subgroup analysis for UC consisted of 23 cohort studies assessing 225 patients with a pooled proportion of clinical remission of 21% (95% CI: 8%-38%), with a high risk of heterogeneity (Cochran's Q, P<0.01, I^2^= 88%).

In meta-analysis of four published RCTs, FMT was significantly more effective than placebo in induction remission of active UC (28% in FMT group versus 9% in placebo groups, OR: 3.67 95% CI: 1.82-7.39,* P*=0.64, I^2^=0%), with no significant risk of heterogeneity between studies. FMT was associated with higher combined clinical and endoscopic remission than placebo.

Regarding the role of FMT in CD, previous meta-analysis showed that the pooled proportion of achieved clinical remission rate was 60.5% (95% CI = 28.4%-85.6%) for CD (P=0.05, I^2^=37%) [48]. Latest meta-analysis showed that the pooled proportion was 52% (95% CI: 31%-72%) for CD, with a moderate risk of heterogeneity [49]. 94 CD patients were enrolled in the current meta-analysis, the pooled proportion of achieved clinical remission was 30% (95% CI: 13%-52%) with a risk of heterogeneity (Cochran's Q, P<0.01, I^2^= 75%), which was lower than that in the previously reported system reviews [16, 41].

There remain major limitations in the available literature of FMT in CD, with the variability of methodology among individual studies and the still limited data; in particular no RCTs data are published to date. Furthermore, unlike UC which is just limited to the mucosal layer, CD usually involves deeper tissues and can be in any region of the gastrointestinal tract. Clinical remission did not correlate with endoscopic outcomes in CD. Efficacy of FMT in CD should be interpreted with caution.

### 4.2. FMT May Be More Efficacious in Severe IBD

Among the 129 mild-moderate IBD patients, 20.9% (27/129) achieved clinical remission, while the clinical remission rate was 32.3% (43/133) in moderate-severe IBD patients. Overall, the pooled proportion of clinical remission rate in mild-moderate patients was 22% (95% CI: 6%-41%, Cochran's Q, I^2^= 84%, P<0.01) with a high risk of heterogeneity, while that in moderate-severe patients was 25% (95% CI: 10%-46%, Cochran's Q, I^2^= 83%, P<0.01). Moderate-severe IBD patients could achieve more significant remission from FMT than mild-moderate patients (*P*=0.037). These interesting findings indicated that FMT might be a potential rescue therapy for refractory IBD, even expected to be an initial standardized treatment for IBD.

### 4.3. Delivery Route Has No Impact on the Efficacy of FMT in IBD

Common upper GI delivery route is nasogastric tube, nasoduodenal tube, or nasojejunum. Common lower GI delivery route is colonoscopy or rectal enemas. Systematic review of FMT for the treatment of CDI demonstrated that colonoscopic delivery had higher rates of success than that of other delivery methods [50].

The optimal FMT route in IBD is uncertain. In the present study, 61% (137/225) UC patients selected the lower GI tract, 19% (42/225) selected the upper GI tract, and 20% (46/225) selected both routes. There was an observed trend towards higher clinical resolution rates by lower GI route compared to upper GI route of delivery of FMT in UC [28% (38/137) versus 14%(6/42)]; however no significance was noted in Chi-Squared analysis (*P*= 0.101). According to the present meta-analysis, the rate of clinical remission of FMT for UC was similar via upper or lower GI delivery route, which was consist with the recently published meta-analysis by Costello et al. [51].

Among the 94 CD patients, 67% (63/94) selected the upper GI tract delivery route, and 21% (20/94) selected the lower GI tract delivery route. The clinical remission rate was 41% (26/63) for upper GI route and 50% (10/20) for lower GI route, and similar results were later obtained from a Chi-Squared analysis with no significance between upper and lower GI delivery method (P=0.492). In addition to efficacy, acceptable delivery method for patient is important to the success of FMT therapy. Delivery route has no impact on the efficacy of FMT in UC and CD; clearly future trials are needed to identify safer, efficacious, and economical mode of FMT delivery.

### 4.4. Donor Selection of FMT in IBD

The optimal donor stool for FMT is still uncertain. In cohorts of patients who have undergone FMT to treat CDI, the choice of donor does not have an important impact on the efficacy of therapy [52]. In the present study, donor stools were from healthy adults or children family members or close friends or volunteers. Among the 319 patients, 134 patients received fecal bacteria from their family members or close friends and 21% (26/134) achieved clinical remission. 79 patients were from healthy anonymous donors, with 37% (29/79) achieving clinical remission. 37 patients were from both family members or friends and healthy volunteers and 69 patients did not report any donor source. This makes it difficult to recommend a particular source of donor and more difficult to analyze the pooled results of the studies. Paramsothy [15] and colleagues tried pooled donor stool FMT in UC, which could provide an increased diversity of microorganisms in the stool suspension than that of a single donor. However, it is not clear whether this theoretical benefit translates to improving the efficacy. Further researches are required to optimize the stool processing, dose of stool, treatment protocols, and so on.

### 4.5. Fresh or Frozen Donor Stool Selection of FMT in IBD

Several studies have compared rates of clinical remission of FMT in treating RCDI with fresh or frozen fecal microbiota. Results showed that neither anaerobic or aerobic stool preparation nor fresh or frozen stool significantly influences the efficacy of FMT in RCDI; frozen FMT had equal effects and risk of adverse events to fresh FMT [53]. Cui et al. [36] reported that fresh fecal microbiota appeared to have higher rate of clinical improvement and clinical remission than that of frozen microbiota for refractory CD, but this research just was a case series. In the present analysis, results were obtained with an observed trend towards higher clinical remission rate of frozen stool FMT than that of fresh stool for UC. Based on current available evidence, the clinical remission rate has no significant difference between fresh and frozen FMT for CD. However, the number of cases analyzed in this analysis was smaller. Further research must be conducted to determine the most effective, standardized mode of donor stool sample preparation, which will also help in better understanding of its mechanism of action.

A potential mechanism for the observed benefits of FMT in the treatment of IBD is its colonization of the recipient's intestine with donor flora [54]. In RCDI, several studies have compared the microbiota composition before and after FMT and have shown that fecal bacterial composition of the recipient was highly similar to that of the donor and was accompanied by resolution of symptoms [55, 56]. In the present system review, most studies mainly compared the changes of flora in the fecal samples before and after FMT and identified the flora by extracting bacterial DNA or sequencing 16s rRNA from stool samples. The changing tendency of flora in IBD after FMT tends to the donor, with increased diversity or richness.

### 4.6. Effects of FMT in Paediatric and Adult IBD

Up to date, published literatures on the effect of FMT in paediatric IBD were sparse and no randomized controlled trials have been published. In the present meta-analysis, the pooled estimate of clinical remission of FMT for paediatric UC was 10% (95% CI: 0%-43%) and 45% for paediatric CD (95% CI: 24%-66%), while it was 26% (95% CI: 10%-48%) for adult UC and 22% (95% CI=3%-52%) for adult CD.

Compared to adult population, paediatric population has a dynamic developing gut microbiome and Crohn's disease and ulcerative colitis typically have a much more aggressive course in the paediatric age group; paediatric IBD phenotype may have a pathophysiology that is distinct from adult-onset IBD [57].

Compared to adults, here is a paucity of data available in children who may differ from adults for many reasons including having a dynamic developing microbiome compared to adults who have a relatively stable microbiome. Paediatric IBD and the paediatric microbiome have several unique features that suggest that microbial based therapies could be particularly effective.

### 4.7. Safety of FMT for IBD

Generally speaking, FMT was tolerable and safe for IBD. Common reported complications after FMT treatment were fever, abdominal pain, bloating, diarrhea, and other adverse reactions such as nasal congestion, vomiting, and sore throat, most of which were self-limiting, lasting generally no more than 24 h. Even though some complications need drug treatment, they can also ease quickly.

Due to the reported short term follow-up evaluation, long-term safety such as effects on immune function and potential infections and so on still need more clinical trials. Therefore, it is particularly important to strictly screen fecal donor and extraction process of fecal bacteria before FMT. To improve security, many researches recommended the use of feces donated from children, parents, spouses, relatives, and friends of patient who have the same living environment. As the genetic characteristics of IBD and intestinal microbial differ individually, the donor screen needs further investigation.

## 5. Conclusions

FMT is an effective and safe therapy for both paediatric and adult IBD; fresh or frozen donor stool, delivery route, and antibiotic pretreatment or not have no impact on the efficacy of FMT in IBD. FMT might be a potential rescue therapy for refractory IBD and it is even expected to be an initial standardized therapy for IBD. However, there is insufficient data on the long-term efficacy and safety of FMT in IBD at present. Further researches, especially the RCT study, are needed to identify safer, efficacious, and economical method of FMT and its potential mechanism.

## Figures and Tables

**Table 1:** Characteristics of the included cohort studies of FMT in IBD.

Study	type	n	Age(y)	Severity	Route	Donor	Fresh/Frozen	Clinical response	Clinical remission	Follow-up (months)
Angelberger et al [18] (2013)	UC	5	22-51	moderate-severe	nasojejunal tube and enema	identified but first degree relatives excluded	Fresh	1	0	3
Kunde et al [19].(2013)	UC	10	7-21	mild-moderate	colonoscopy	family members or close friends	Fresh	6	3	1
Kump et al [20].(2013)	UC	6	17-52	moderate-severe	enema	unrelated	Frozen	2	0	3
Cui et al [21] a (2015)	UC	15	11-48	moderate-severe	gastroscope	healthy children (patient relatives or friends)	Frozen	12	4	4-72
Damman et al [22] (2015)	UC	7	27-61	mild-moderate	colonoscopy	family members or close friends	Fresh	1	1	3
Ren et al [23] (2015)	UC	7	17-66	severe	Colonoscopy and gastroscopy	family members or unrelated	Fresh	7	7	1-7
Scaldaferri et al [24].(2015)	UC	8	30-44	mild-moderate	colonoscopy	unrelated	NR	4	3	3
Wei al et [25] a(2015)	UC/CD	14	16-70	mild-moderate		unrelated	Frozen	9	6	1
Suskind et al [26] a.(2015)	UC	4	13-16	mild-moderate	nasogastric	NR	Fresh	0	0	3
Vermeire et al [27].(2016)	UC/CD	14(8/6)	28-53	moderate-severe, refractory, failed immunotherapy and anti-TNF	Nasojejunal or rectal	family members or close friends	Fresh	2	2	2
Nishida et al [28].(2017)	UC	41		mild-moderate	colonoscopy	family members	Fresh	11	0	2
Wei et al [29] b (2016)	UC	20	18-70	mild-moderate	colonoscopy	unrelated	Fresh	13	7	3
Ishikawa et al [30] (2017)	UC	17	21-65	mild-to-severe active	colonoscopy	family members	Fresh	14	9	1
Zhang et al [31] (2016)	UC	19	19-60	moderate-severe	gastroscope	NR	Fresh	11	2	≥3
Karakan et al [32].(2016)	UC	14	NR	Steroid-dependent	colonoscopy	NR	NR	11	6	3-18
Goyal et al [33].(2016)	UC/CD	16(12/4)	<18	mild-moderate	gastroscope	NR	Fresh	5	2	6
Kellermayer et al [34] (2015)	UC	3	14-16	immunotherapy dependent	colonoscopy and enema	unrelated	Frozen	3	3	3
Karolewska-Bochenek et al [35] (2015)	UC	4	10-17	mild-moderate	gastroscope	unrelated	NR	4	0	1
Cui et al [36] b.(2015)	CD	30	15-71	moderate-severe	gastroscope	family members or close friends or unrelated	23 Fresh 7 Frozen	20	18	6-15
Suskind et al [37] b (2015)	CD	9	12-19	mild-moderate	nasogastric	family members(parents)	Fresh	7	5	3
Vaughn et al [38].(2016)	CD	19		active	colonoscopy	unrelated		11	10	6.5
Zhang et al [39].(2013)	CD	16	14-63	refractory	gastroscope	NR	Frozen	12	3	1
Alka-Goyal etal [40],(2018)	UC/CD	21(14/7)	8-21	refractory	colonoscopy and gastroscopy	family members or close friends	Fresh	6	2	6

UC: ulcerative colitis; CD: Crohn's disease; NR: no report.

**Table 2:** Prevalence in different subgroups.

Stratification group	Number of studies	Number	Events	*I* ^*2*^	*p* value	Prevalence	95% CI
Current prevalence	23	319	93	86%	<0.01	0.27	0.15-0.41

**Subtype**							
UC	19	225	53	88%	<0.01	0.21	0.08-0.38
CD	8	94	40	75%	<0.01	0.3	0.13-0.52

**Populations**							
Paediatric UC	6	47	6	85%	<0.01	0.1	0.00-0.43
Adult UC	13	178	45	88%	<0.01	0.26	0.10-0.48
Paediatric CD	3	20	9	0%	0.53	0.45	0.24-0.66
Adult CD	5	74	31	85%	<0.01	0.22	0.03-0.52

**Preparation of inoculum**							
Fresh for UC	12	164	31	89%	<0.01	0.15	0.03-0.37
Frozen for UC	4	35	13	87%	<0.01	0.42	0.05-0.86
Fresh for CD	5	49	24	78%	<0.01	0.36	0.10-0.68
Frozen for CD	4	45	16	69%	0.02	0.28	0.07-0.55

**Route of administration**							
Upper GI for UC	4	42	6	51%	0.10	0.08	0.00-0.26
Lower GI for UC	10	137	38	88%	<0.01	0.31	0.11-0.57
Upper GI for CD	5	63	26	83%	<0.01	0.23	0.02-0.55
Lower GI for CD	2	20	10	60%	0.11	0.27	0.00-0.93

**Disease severity**							
Mild-moderate	9	129	27	84%	<0.01	0.20	0.06-0.41
Moderate-severe	11	133	45	83%	<0.01	0.30	0.10-0.54

**pre-antibiotic treatment or not**							
Pretreatment	7	85	29	73%	<0.01	0.25	0.09-0.46
Un-pretreatment	16	234	64	89%	<0.01	0.28	0.13-0.47

**Table 3:** RCTs of FMT in UC.

	Costello et al [41].	Moayyedi et al [14].	Paramsothy et al [15].	Rossen et al [16].
Number	73(38 FMT/35 placebo)	75(38 FMT/37 placebo)	81(41 FMT/40 placebo)	48(23 FMT/25 placebo)
Severity	mild-moderate	mild-moderate	mild-moderate	mild-moderate
Route	colonoscopy and enema	enema	colonoscopy and enema	nasoduodenal
Placebo	Autologous stool	water	water	Autologous stool
Donor	unrelated	unrelated	unrelated	unrelated
Fresh vs Frozen	Frozen	frozen and fresh	Frozen	Fresh
Frequency	3	6	40(5x/week for 8 weeks)	2
Follow-up	8 weeks	6 weeks	8 weeks	12 weeks
Remission	12/38(FMT)	9/38(FMT)	11/41(FMT)	7/23(FMT)
FMT/placebo	3/35(placebo)	2/37(placebo)	3/40(placebo)	5/25(placebo)
Response	21/38(FMT)	15/38(FMT)	22/41(FMT)	11/23(FMT)
FMT/placebo	7/35(placebo)	9/37(placebo)	9/40(placebo)	13/25(placebo)

## Supplementary Materials

Supplementary Materials Supplement-Figure 1: Forest plot for the pooled proportion of remission of FMT for IBD patients in cohort studies. Supplement-Figure 2: Forest plot for the pooled proportion of remission of FMT for UC (2a) and CD (2b) patients in cohort studies. Supplement-Figure 3: Forest plot for clinical remission (3a) and clinical response (3b) of FMT for UC patients in randomized controlled trials.
