# Supplementary material for: Protocol for Fecal Microbiota Transplantation in Inflammatory Bowel Disease: A Systematic Review and Meta-Analysis
Source: Biomed Res Int. 2018 Sep 13;2018:8941340. doi: 10.1155/2018/8941340 (PMC6158944; doi:10.1155/2018/8941340)
Supplement: Supplementary Materials — Supplement-Figure 1: Forest plot for the pooled proportion of remission of FMT for IBD patients in cohort studies. Supplement-Figure 2: Forest plot for the pooled proportion of remission of FMT for UC (2a) and CD (2b) patients in cohort studies. Supplement-Figure 3: Forest plot for clinical remission (3a) and clinical response (3b) of FMT for UC patients in randomized controlled trials. [file 8941340.f1.zip › 8941340.f3.doc]

Supplement-figure 3 Forest plot for clinical remission (3a) and clinical response (3b) of FMT for UC patients in randomised controlled trials
